# Reproductive Potential of Field-collected Populations of *Cimex lectularius* L. and the Cost of Traumatic Insemination

**DOI:** 10.3390/insects2030326

**Published:** 2011-07-05

**Authors:** Andrea M. Polanco, Dini M. Miller, Carlyle C. Brewster

**Affiliations:** Department of Entomology, Virginia Tech, 216A Price Hall, Blacksburg, VA 24061, USA; E-Mails: apolanco@vt.edu (A.M.P.); carlyleb@vt.edu (C.C.B)

**Keywords:** bed bugs, egg production, multiple matings, fecundity reduction

## Abstract

Egg production was compared among three field-collected bed bug strains over the course of 13 feeding/oviposition cycles, each of which lasted ∼10 days. No significant differences were found among bed bug strains in the mean number of eggs/female/day (∼1.0 egg). However, significant differences were found among strains in their patterns of egg production throughout the study period. Specifically, differences were observed in the timing of peak egg production and the rapidity of egg production decline among the three strains. Egg production was also quantified for female bed bugs that were subjected to single or multiple traumatic insemination events over a period of six feeding/oviposition cycles. Significant differences were found in egg production between females exposed to single and multiple inseminations. Females mated only once produced 83.8 ± 4.5 (mean ± SE) eggs over six feeding cycles. Females exposed to multiple inseminations produced 61.0 ± 3.1 eggs, indicating that multiple traumatic inseminations may reduce female fecundity by as much as 27%. This study is the first to suggest that, in a new infestation (first ∼6 weeks), a solitary, singly-mated female with access to regular blood meals is capable of producing greater numbers of offspring than the same female in the presence of a male.

## 1. Introduction

Within the family Cimicidae, there are three species that are human ectoparasites: *Cimex hemipterus*, the predominant pest of tropical latitudes; *Leptocimex boueti*, found in West Africa; and *C. lectularius*, the dominant pest in temperate regions, including the U.S. *Cimex lectularius* is an obligate hematophage, which has five nymphal instars, each requiring a blood meal to survive and develop into the next life stage. The frequency of feeding, which is dependent on host availability, directly affects the bed bug nymphal development times. If a host is not present, bed bug nymphs cannot feed or develop to the next instar. Adult male and female bed bugs also require multiple blood meals to reproduce [1]. Females must consume a blood meal in order to produce eggs. Therefore, the frequency of feeding by a *C. lectularius* female directly affects how many eggs she will produce in her lifetime. In addition, the size of the blood meal taken by a female will influence her egg production. The larger the female the larger the blood meal she can take. Consequently, a large female produces more eggs than a smaller female [1].

Several studies conducted prior to the 1950s quantified the reproductive potential of *C. lectularius* in an attempt to predict population growth [2,3]. Johnson [3] found that at 23 °C, females began to produce eggs five to six days after feeding. These females would continue to produce eggs for approximately six days until 6–10 eggs had been produced from the initial blood meal. Johnson [3] also found that egg production was continuous when bed bugs had access to regular blood meals. In fact, egg production increased from an average of 3 eggs/week after the first feeding (week one) to over 8 eggs/week after week four. With regular access to blood meals, Johnson [3] recorded females producing over 5 eggs/week for a total of 18 weeks. In a similar study, Davis [2] reported that females held at 27 °C produced an average of 3 eggs/day for 11 days after a single blood meal. The egg production numbers reported by Johnson [3] and Davis [2] appear to be considerably lower than those reported earlier by Hase [4], who observed a maximum of 12 eggs being produced by a single female in one day; and Titschack [5], who reported 541 eggs being produced by a single female over her lifetime. Overall, the results of egg production studies have been variable, but this variability suggests that there are a number of factors that may influence an individual female's egg production, such as size of the female, number of matings, frequency of blood meals, and insecticide resistance.

Several studies have suggested that the cimicid method of copulation, termed “traumatic insemination” influences female egg production [1]. The male uses his reproductive organ (paramere), to pierce the female through her abdominal wall. He then releases his sperm into the female ectospermalege [6]. Multiple traumatic inseminations are known to be costly for the female, reducing her fitness in terms of life span and reproduction [7]. In fact, females have been killed when placed in experimental containers with more than six males [1]. While this type of extra-genital insemination is a relatively rare phenomenon in the animal kingdom, it has been studied in several invertebrate taxa [8], including bed bugs [7–10].

Stutt and Siva-Jothy [8] studied seven different bed bug populations to quantify the reproductive cost of multiple matings. Stutt and Siva-Jothy [8] found that insemination once every four blood meals was sufficient to maintain reproductive success in a population of bed bugs. However, male bed bugs are known to be sexually active after feeding, and the rate of copulation in the natural environment has been estimated to be up to 20 times more than is necessary to maintain maximum fertility [1]. Siva-Jothy and Stutt [10] suggested that the energy requirements to heal the abdominal wounds caused by excessive copulation might cost the female bed bug as much as a 24% reduction in reproductive output.

No comprehensive studies on bed bug egg production have been conducted in over 45 years. It is possible that populations of bed bugs currently infesting the United States have been subjected to selection pressures (e.g., pesticide exposure) that did not exist when the previous studies by Hase [4], Johnson [3], or Davis [2] were conducted. Therefore, the reproductive potential suggested in these prior studies may not be representative of modern populations infesting the U.S. today. In addition, we do not fully understand the impact of multiple matings on a female's reproductive potential. For example, if we released a female from the pressure of multiple matings, would her reproductive potential increase as suggested by Stutt and Siva-Jothy [8]?

The purpose of this study was to quantify the egg production potential of field-collected bed bug strains over the course of multiple blood meals (until onset of adult female mortality). In addition, the number of eggs produced by females subjected to a single copulation was compared with those exposed to repeated traumatic inseminations.

## 2. Materials and Methods

### 2.1. Bed Bug Rearing

Two field strains of bed bugs, Richmond (RI) and Nottingham Green (NG) were collected from apartments in Richmond, VA in September 2008 and July 2009, respectively. Another field strain, Epic Center (EC) was collected in June 2008 from hotel rooms in Cincinnati, OH. The bed bug colonies were reared in plastic jars covered at one end with cloth mesh, and sealed on the opposite end with a plastic lid. Two pieces of cardboard were placed inside of the jars so that bed bugs could crawl up the cardboard and stick their mouth-parts through the cloth mesh to feed on an artificial feeder. An artificial feeder using circulating hot water maintained a diet of chicken blood at 35.5 °C. Bed bugs were fed once a week on chicken blood formulated with sodium citrate (Lampire Biological Laboratories, Pipersville, PA, USA) as an anti-coagulant. Between feedings, bed bugs were stored in an environmental chamber at temperatures between 26.1–26.5 °C, 68.9% RH and a photoperiod of 12:12 h, L:D. These conditions were similar to the conditions at which Johnson [3] evaluated bed bug populations. All bed bug colonies were maintained in the Dodson Urban Pest Management Laboratory at Virginia Tech, Blacksburg, VA, USA.

### 2.2. Evaluation of Life-long Egg Production Potential (Multiple Matings)

The three field-collected bed bug strains (RI, EC, NG) were evaluated to determine their life-long egg production potential. Twenty male and female fifth instars were selected from each strain and allowed to feed and then molt to adulthood. In order to mimic a natural host that bed bugs would find in the field, fifth instars were allowed to feed on a human volunteer. The process of feeding the fifth instars was conducted by inverting the mesh-covered opening of the rearing jars against the arm of a human, as approved by the Virginia Tech Institutional Review Board (IRB 06-165). Once the bed bug nymphs molted into adults, they were again fed on human blood, as described above. After feeding, pairs of adults (1 male: 1 female) were placed in individual Petri dishes (Fisher brand 60 × 15 mm) containing a single piece of filter paper (Whatman 42.5 mm) and left to produce eggs. The filter paper in each Petri dish was replaced daily and the number of eggs on the paper was counted and recorded until egg laying ceased. Similar to the Johnson [3] study, an additional human blood meal was offered on the second or third day after the cessation of oviposition. Egg laying was continuously recorded for 13 feedings/oviposition cycles or until the adult female died.

### 2.3. Comparison of Fecundity: Single *versus* Multiple Matings

Virgin female bed bugs from the RI field strain were collected on the day after adult eclosion. Females were then allocated at random to one of two experimental treatments. In the first treatment, 20 females were fed to repletion as described above; after feeding each female was placed inside a Petri dish (Fisher brand 60 × 15 mm) containing a single filter paper (Whatman 42.5 mm) and a virgin male. After copulation, the male was removed and the female remained isolated in the Petri dish. The mated females were fed every 11 days and the number of eggs produced was counted each week for six feeding/oviposition cycles. A second group of virgin females was fed and placed individually in Petri dishes with a single fed virgin male. The pair was allowed to copulate repeatedly throughout the test period. The mating couples were fed every 11 days and the number of eggs produced for each of six feeding/oviposition cycles was counted. Only fertile eggs were counted in each treatment.

### 2.4. Statistical Analysis

The egg production data for the three bed bug strains were transformed $\left(\sqrt{y+0.5}\right)$ and analyzed using repeated measures MANOVA [11,12]. The repeated measures MANOVA examined two effects, a between-subject effect of the main factor (bed bug strain) and a within-subject effect that included time (feeding/oviposition cycle or days after feeding) as the repeated measures factor. For the analysis of the within-subject effect, significant time (feeding/oviposition cycle or days after feeding) × main factor interactions were examined to determine how the patterns of oviposition across each of the time factors differed with respect to the three bed bug strains [12]. In all analyses where significant differences were detected (α = 0.05), multiple comparisons of the mean responses of the factor levels were carried out by orthogonal contrast [12]. All statistical analyses were carried out using JMP 8.0 (SAS Institute Inc., Cary, NC, USA).

In addition to the above analyses, we fitted the data on the mean number of eggs/female for each of the days within the feeding cycles to a Ricker function [13]

(1) Y=aXexp(−bX)

where *Y* is the predicted mean number of eggs/female/day, *X* is the day within the feeding cycles, *a* is the maximal egg production rate throughout the observation period, and *b* is a constant representing the extent of decline in egg production over successive days within the feeding cycles [13]. The parameters of the model were estimated using nonlinear least-squares in TableCurve 2D 5.01 (SYSTAT Software Inc., Richmond, CA, USA).

When evaluating the egg production from females mated singly and females exposed to multiple copulations, the overall mean number of eggs oviposited by each test group of females was calculated. Significant differences in mean egg production between single-mated and multiple-mated females were determined using repeated measures MANOVA. The egg production data for each test group were transformed $\left(\sqrt{y+0.5}\right)$ prior to analysis.

## 3. Results

### 3.1. Evaluation of Lifelong Egg Production Potential

Egg production within the three bed strains (RI, NG, and EC) was observed over the course of 13 feeding/oviposition cycles, each of which lasted ∼10 days. At the end of the study period, 17, 13, and 15 females (out of an original 20) from the RI, NG, and EC strains, respectively, were dead.

The overall mean (±SE) number of eggs oviposited per female during all of the 13 feeding/oviposition cycles was 131.9 (±23.7), 153.9 (±19.8), and 155.7 (±21.0) for the RI, NG, and EC strains, respectively. No significant differences were found in the mean number of eggs/female/feeding cycle for the RI (8.8 ± 0.98 SE), NG (9.3 ± 0.71 SE), and EC 8.9 (±0.80 SE) strains, based on the overlapping confidence intervals.

**Figure 1:**
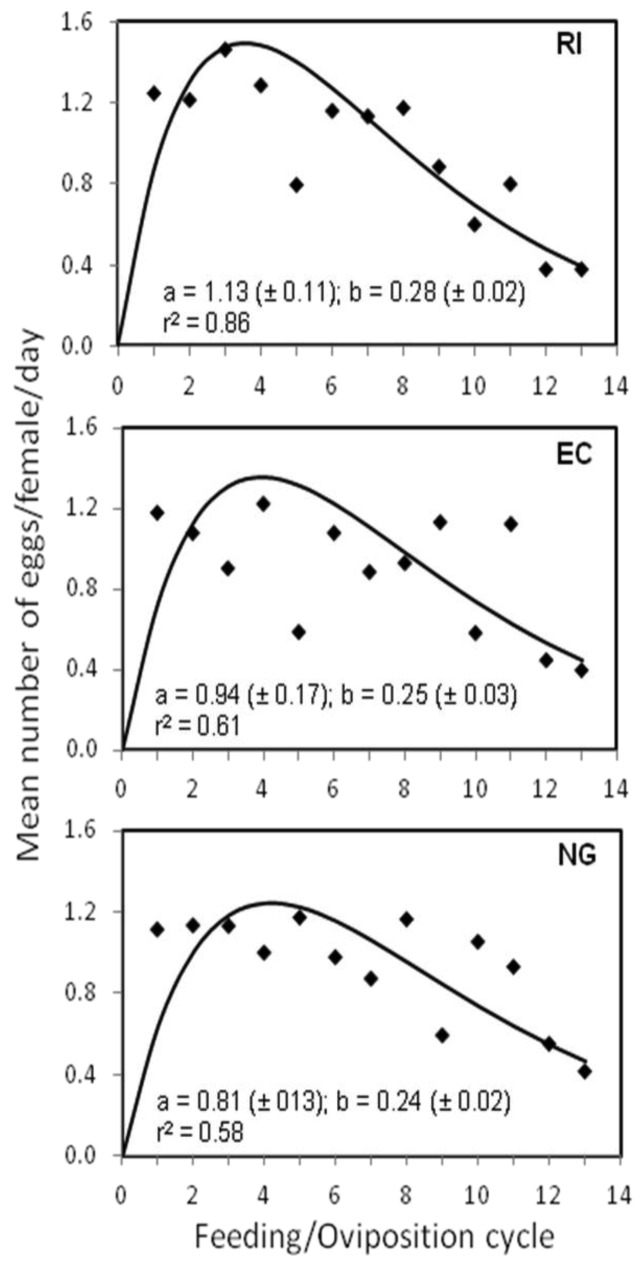
Mean number of eggs/female/day (filled diamonds) and fit of the model [*Y* = *aX* exp(−*bX*), solid line] to these data for three strains of the common bed bug (Richmond-RI, Epic Center-EC, and Nottingham Green-NG) over the course of 13 feeding/oviposition cycles. Shown are estimates of the model parameters (±SE).

The results of the repeated measures MANOVA showed that there were also no significant differences among the three bed bug strains in the mean number of eggs/female/day (*F*_2, 141_ = 0.492, *p* > 0.05). Individual females from the RI, NG, and EC bed bug strains produced an average of 0.96 (±0.10 SE), 0.93 (±0.07 SE), and 0.89 (±0.08 SE) eggs/day, respectively, throughout the 13 feeding/oviposition cycles. However, significant differences (*F*_24, 1692_ = 3.302, *p* < 0.0001) were found among the three strains in the patterns of oviposition per female per day across the 13 feeding cycles, with the RI and EC strains having similar egg-laying patterns that were statistically different from that of the NG strain (Figure 1). The patterns of oviposition are clearer by looking at the fit of equation 1 to the egg production data (Figure 1). There was a rapid increase in oviposition during the early feeding cycles for all three strains followed by a decline in egg production with subsequent feedings. However, the peak in daily oviposition by individual females occurred during the third and fourth feeding cycles for the RI and EC strains, but during the fifth feeding cycle for the NG strain (Figure 1). The RI strain also had the greatest maximal rate of increase (*a* = 1.13) and decline (*b* = 0.28) in daily egg production followed by the EC and NG strains.

**Figure 2:**
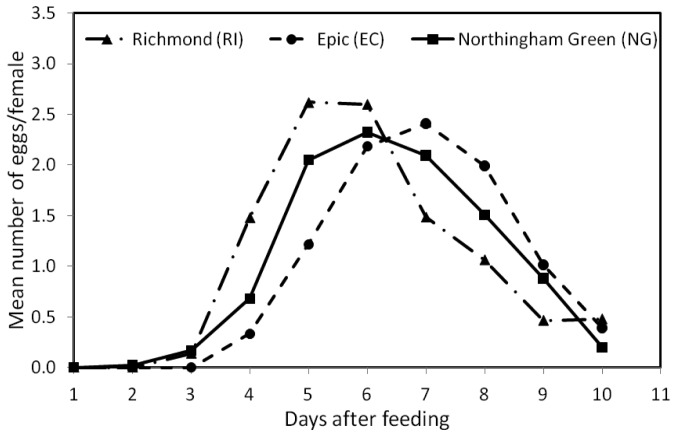
Mean number of eggs produced by females of three bed bug stains (Richmond-RI, Epic Center-EC, and Nottingham Green-NG) on specific days after feeding during 13 feeding/oviposition cycles. The three strains had significantly different (*p* < 0.05) egg production patterns across days after feeding.

Figure 2 shows the mean number of eggs per female at each of the 10 days within the 13 feeding/oviposition cycles. The overall mean (±SE) number of eggs/female was similar for the RI (1.03 ± 0.32 SE) and NG (0.99 ± 0.29 SE) strains, but significantly different from that of the EC (0.95 ± 0.30 SE) strain (*F*_2, 479_ = 3.603, *p* < 0.05). The patterns of oviposition across days after feeding for the three strains were also significantly different (*F*_18, 4311_ = 15.08, *p* < 0.0001) (Figure 2). During the 10-day period, EC females began egg production 2 days later than NG females and 1 day later than the RI females, or four days after feeding (Figure 2). Peak egg production for the EC strain also occurred at day 7, which was ∼2 days later than the other two strains.

### 3.2. Comparison of Fecundity: Single *versus* Multiple Matings

**Figure 3:**
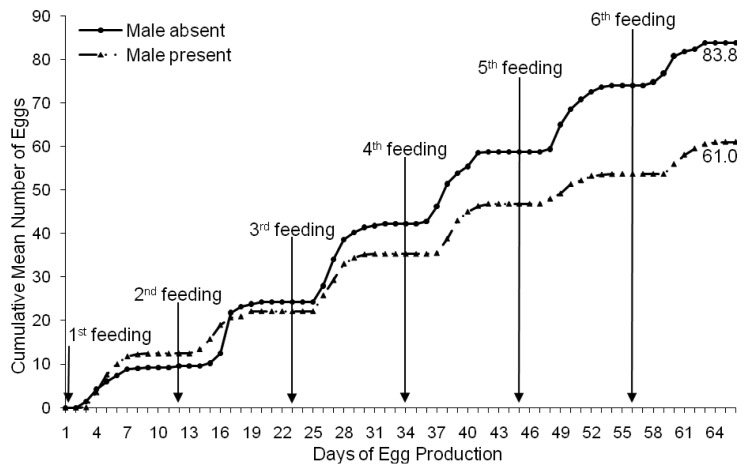
Cumulative mean number of eggs produced by 20 females of the RI bed bug strain mated once or exposed to multiple matings over the course of six feeding/oviposition cycles. Bed bugs were fed on the days denoted by arrows.

Females isolated from males after a single mating produced an average of 83.8 (±4.5 SE) eggs per female over six feeding/oviposition cycles (Figure 3). Females exposed to multiple copulations produced significantly less eggs on average (61.0 ± 3.1 SE eggs/female) over the six feeding/oviposition cycles (*F*_1, 438_ = 4.945, *p* < 0.05). Singly-mated females produced 27% more eggs than females exposed to multiple matings. The egg production patterns of singly-mated females and females exposed to multiple matings were also significantly different (*F*_5, 2190_ = 10.04, *p* < 0.0001).

## 4. Discussion

The females from all three field-collected strains (EC, NG, and RI) produced an overall average of 147 eggs during their adult lifespan, or ∼1.0 eggs/female/day during each of the 13 feeding/oviposition cycles. These numbers were relatively low when compared to some studies conducted prior to 1940 that quantified bed bug egg production (e.g., Hase [4], Titschack [5]). Egg production per female per day in our study, however, was consistent with what Johnson [3], and Bell and Schaefer [14] found for females reared on rabbit blood (∼1.2 and 1.5 eggs per female per day, respectively). Some studies have shown humans to be a relatively poor food source in terms of egg production, when compared to mouse and fowl [15], and rabbits [16]; however, the conditions and results of those studies were quite variable. Neither Johnson [3] nor Bell and Schaefer [14] specifically report life-long egg production per female.

In this study, bed bug females from the three field strains (RI, EC, and NG) produced eggs for a period of 10 days after a blood meal (Figure 2). These results were similar to those reported by Johnson [17] (8–10 days egg production after feeding) for the same species. We also found that the three strains had similar overall egg production, but differed in their patterns of oviposition across time. Therefore, we might expect populations of different bed bug strains in their natural environment with comparable numbers of females to also vary in their egg-laying patterns, but with similar overall egg production capacities.

In spite of the different patterns of egg production observed in this study, we note that, in general, female bed bugs started producing eggs on the third day after a blood meal (Figure 2), a result which was also reported by Johnson [17] for bed bugs held at 27 °C. Similarly, How and Lee [18] found that egg production in *C. hemipterus* began 2–5 days after a blood meal. We also found that maximum egg production occurred during the first four feeding/oviposition cycles (Figure 1), which concurs with Johnson [17]. How and Lee [18], however, reported that egg production in *C. hemipterus* peaked at the second and third oviposition cycle.

In our study, egg production was observed to decrease substantially after about the 10th feeding/oviposition cycle (Figure 1). Johnson [17] also observed that female bed bugs became less fertile with age. The fact that the majority of the female bed bugs in our study were dead by the end of the 13th feeding/oviposition cycle (∼130 days) suggests that fertility was reduced due to female morbidity upon approaching the end of their lifespan. However, our study also suggests that the repeated copulation may have contributed to female morbidity and subsequent reduction in egg production over time.

How and Lee [18] found no significant differences in the number of eggs produced by six different field strains of tropical bed bugs. Likewise, we found no significant differences in egg production among our field strains. While we know little about the egg production potential of bed bug populations across the United States, we do have some idea of their physiology. For example, recent studies have led us to conclude that pyrethroid resistance is highly prevalent in U.S. populations. Our laboratory studies over the last five years (2005–2010) have documented a range in field strain susceptibility to insecticides. All three of the bed bug field strains used in this study have been documented to have high levels of pyrethroid resistance [19,20]. The Richmond strain has also been documented to have large numbers of upregulated genes that are known to code for detoxification enzymes [21]. Insecticide resistance, specifically resistance resulting from enhanced detoxification enzyme activity, is known to reduce fecundity and survivorship potential. Several studies have demonstrated that resistant insect populations typically produce shorter adult life spans and fewer offspring than susceptible populations [3]. However, the reproductive potential of many more populations will need to be quantified to obtain some measure of the variability (with regard to fecundity) that might exist in modern bed bug populations due to different levels of insecticide resistance.

In this study, we did not quantify the number of copulations that took place after each blood meal. However, Stutt and Siva-Jothy [8] found that females copulated an average of five times within 36 hours of taking a blood meal. If females copulated at this rate (five matings per blood meal) throughout the test, they may have copulated as many as 65 times. If this were the case, females that were mated repeatedly, would have to recover from as many as 65 integumental wounds. We would expect that this large number of wounds would shorten the female's lifespan, and thus, reduce egg production towards the end of her life. In fact, Stutt and Siva-Jothy [8] indicated that the life length of repeatedly mated females was significantly shorter (∼37 days) than females in low-mating groups.

Stutt and Siva-Jothy [8] reported that the repeated traumatic inseminations resulted in reduced longevity and subsequent reproductive output in female bed bugs by 24%. Specifically, the Stutt and Siva-Jothy [8] study concluded that the repeated inseminations reduced the average female life length from 147 to 110 days thus reducing the reproductive potential in females subjected to repeated matings from an average of 294 to 224 eggs per lifetime.

Our study differed from the Stutt and Siva-Jothy [8] study in that female egg production was not compared over the entire life length but during the prime egg production period prior to the natural decline in egg production that would occur at the sixth feeding cycle with the onset of morbidity. In our study, individual female bed bugs were exposed to a single copulation during the first feeding cycle, while others were exposed to a male bed bug continuously throughout six feeding cycles. We found that females that were isolated from males after insemination produced a significantly greater number of eggs during the six feeding cycles than those that were exposed to repeated traumatic inseminations. In fact, this is the first study to document that egg production in females not subjected to repeated inseminations is ∼27% greater than females that had constant access to males. Our results indicate that a single mated female bed bug with access to regular blood meals would actually be more fit for starting a new infestation alone than if a male were present.

## 5. Conclusions

Our study showed that individual female bed bugs from the field-collected populations produced an average of ∼147 eggs (1.0 eggs/female/day) over a period of 13 feeding/oviposition cycles. Tests were terminated after 13 cycles due to mortality of females. There were no significant differences in the number of eggs produced by females from the different field-collected strains (*p* > 0.05). However, the egg production pattern (peak production and subsequent decline) over the test period was found to be similar for the RI and EC strains, but significantly different for the NG bed bug strain. Our results also demonstrated that female bed bugs exposed to a single mating event produced 27% more eggs than females exposed to multiple matings. Therefore, a mated female would be expected to lay more eggs in the absence of males, than she would in the presence of a single male or multiple males. However, singly-mated females would eventually run out of sperm and would require subsequent matings.
